# Cells Versus Cell-Derived Signals in Cardiac Regenerative Therapy: A Comparative Analysis of Mechanisms and Clinical Evidence

**DOI:** 10.3390/cells14211674

**Published:** 2025-10-27

**Authors:** Julia Soczyńska, Wiktor Gawełczyk, Krzysztof Majcherczyk, Julia Rydzek, Adrian Muzyka, Mateusz Żołyniak, Sławomir Woźniak

**Affiliations:** 1 Student Scientific Society Anatomia-Klinika Nauka, Division of Anatomy, Department of Human Morphology and Embryology, Wroclaw Medical University, 50-367 Wroclaw, Poland; wiktor.gawelczyk@student.umw.edu.pl (W.G.); krzysztof.majcherczyk@student.umw.edu.pl (K.M.); julia.rydzek@student.umw.edu.pl (J.R.); adrian.muzyka@student.umw.edu.pl (A.M.); mateusz.zolyniak@student.umw.edu.pl (M.Ż.); 2 Division of Anatomy, Department of Human Morphology and Embryology, Wroclaw Medical University, 50-367 Wroclaw, Poland; slawomir.wozniak@umw.edu.pl

**Keywords:** cardiomyocyte, cell therapy, cell-derived signals

## Abstract

Heart failure (HF) and other cardiac pathologies represent leading causes of hospitalization and mortality worldwide, underscoring the urgent need for effective regenerative therapies. In recent years, considerable research has focused on developing cell-based therapeutic strategies, with stem cells receiving particular attention. Approaches that harness cellular signaling pathways have also been investigated. Experimental studies conducted in both animal models and human subjects have demonstrated that cell-based therapies hold remarkable potential, showing efficacy through improvements in cardiac function, patient quality of life, and overall safety. Clinical data concerning therapies based on cellular signals, while sometimes inconclusive, often yield outcomes comparable to or even superior to those of cell-based interventions. Nonetheless, both approaches face substantial challenges, including the need to ensure reproducibility of results, standardization of therapeutic product preparation, and addressing ethical and regulatory considerations. To translate these promising strategies into clinical practice, a greater number of large-scale, multicenter, and diverse clinical trials will be required.

## 1. Introduction

Cardiac regenerative therapies, including both cell transplantation and the use of cell-derived signals (e.g., exosomes), are being intensively studied as potential treatments for HF and post-infarction damage. Over the past two decades, more than 200 clinical trials involving thousands of patients have been conducted, focusing mainly on stem cells such as mesenchymal stem cells (MSCs) and progenitor cells. Cell therapies are considered safe, but their efficacy is moderate; e.g., improvement in left ventricular ejection fraction (LVEF) is typically 2–5% compared to placebo [1,2,3,4]. A key limitation is the very low survival rate of transplanted cells: after 2 h, approximately 5% of cells remain in the heart, and after 20 h, only 1% [5,6]. These results suggest that direct integration of cells with heart tissue is limited, and therapeutic effects result mainly from paracrine action, i.e., the secretion of signaling factors such as exosomes and microRNAs by cells [5,7,8,9,10]. Animal models have shown that administering exosomes alone can lead to comparable or even better improvement in heart function than cell transplantation, but the number of large clinical trials involving humans is still small, and there is a lack of precise data on the effectiveness of these therapies [7,8,9,10]. In the cellular approach to the heart, stem cells (e.g., MSCs, progenitor cells, cells derived from pluripotent sources) are introduced into the heart, which can directly differentiate into new cardiomyocytes and vascular cells, support repair by integrating with heart tissue, and activate endogenous repair mechanisms through the secretion of paracrine factors [1,6,8,11,12]. Therapies based on cell-derived signals, especially exosomes and other extracellular vesicles that transport proteins, lipids, and microRNAs that regulate repair processes, stimulate angiogenesis, cell proliferation, inhibit apoptosis and fibrosis, can be administered directly, bypassing problems with cell survival and integration [7,10,13,14,15,16,17,18]. In the context of cell therapies, one of the key limitations remains low survival rates and limited retention of cells at the target site after administration. It is estimated that only 2–5% of transplanted cells effectively reach the damaged heart muscle and remain in its structure, which significantly reduces their regenerative potential [6,12,19]. In addition, cells derived from pluripotent sources often exhibit an immature phenotype, poor ability to integrate into cardiac tissue, and pose a risk of inducing arrhythmia [4,6]. Issues of immunogenicity and potential malignant transformation also remain significant problems, especially when allogeneic or pluripotent cells are used, which may lead to rejection reactions or uncontrolled proliferation [6,19,20]. The clinical efficacy of cell therapies varies greatly depending on a number of factors, such as the type of cells used, their dose, route of administration, and the individual clinical condition of the patient [12,19,21,22]. Therapies using cell-derived signals, including exosomes, also face significant difficulties. Key challenges include a lack of standardization in isolation and production processes, as the composition, quality, and effective dose of exosomes depend on the cell source and the physiological condition of the donor [15,20,23]. These factors influence the repeatability and predictability of therapeutic effects [15,24]. Current knowledge about the efficacy of these approaches comes mainly from preclinical studies, with no large randomized clinical trials involving patients [13,15,25]. Furthermore, the molecular mechanisms underlying cellular signaling remain poorly understood, which significantly hinders the process of optimizing therapy and predicting its clinical effects [13,24]. 

**Figure 1:**
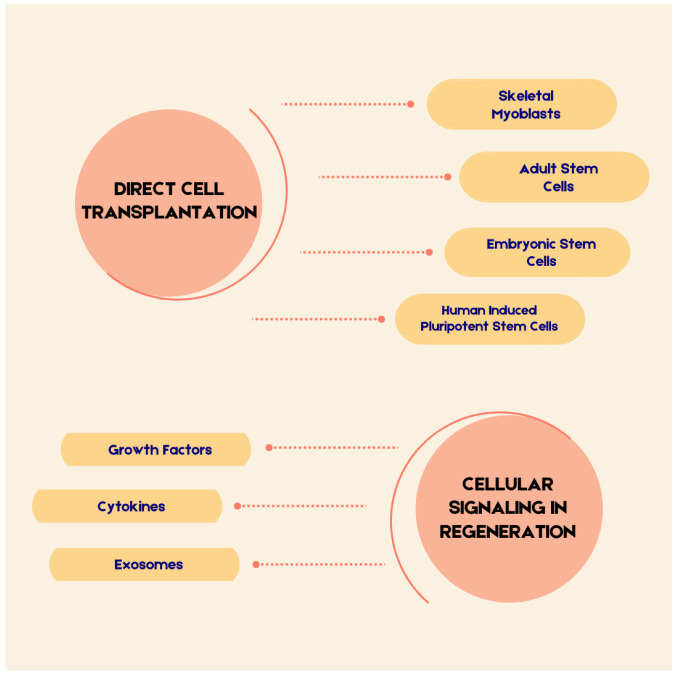
The Elements Discussed in the Text.

Figure 1 presents the key aspects that will be discussed in this review.

## 2. Mechanisms of Cardiac Muscle Regeneration

### 2.1. Structure of the Cardiac Muscle Cell in Health and Disease

The heart is a four-chambered organ that plays a central role in maintaining systemic circulation. It serves as the primary pump of the circulatory system, yet its significance extends beyond a purely mechanical function [26]. On average, the heart beats approximately 100,000 times per day, amounting to roughly 2.5 billion beats over the course of a normal lifespan [27]. To sustain its cyclical activity, the cardiac conduction system orchestrates the heart’s rhythm in cooperation with the myocardium. The force of contraction, which depends on the length of the contractile element of cardiac muscle, can be modulated indirectly through sympathetic impulses or by the activity of mediators such as adrenaline [26,28]. These mediators act on receptors, producing distinct effects depending on the isoform involved. For example, β1-adrenergic receptors (β1AR) are associated with chronotropy and lusitropy, whereas β2-adrenergic receptors (β2AR) exert no influence on lusitropy and only modestly contribute to chronotropy [29].

The high degree of specialization and cooperation among all components is crucial for normal function, but it also imposes certain limitations. In mammals, including humans, mature hearts exhibit insufficient capacity for de novo cardiomyocyte generation, despite the presence of this ability during fetal development [30]. Furthermore, there are reports of adverse effects on cardiac function induced by artificial interventions, such as the delivery of specific proteins (e.g., Yap—Yes-associated protein) or microRNAs (e.g., miR-199a) [30,31].

#### 2.1.1. Physiological Structure

Depending on their location, cardiomyocytes can be classified as atrial, ventricular, or conduction system cells [32]. The literature reports that cardiomyocytes are cylindrical in shape, with diameters ranging from 17 to 25 μm and lengths between 60 and 140 μm [33]. Cardiomyocytes are interconnected via intercalated discs, enabling both electrical and mechanical coupling [34]. According to Keepers et al., defining characteristics of cardiomyocytes include their function, localization, origin, size, centrally positioned nucleus, and abundant sarcoplasm [35]. The spatial organization of organelles within cardiomyocytes is highly structured and functionally significant. For instance, mitochondria, which occupy approximately 40% of the volume of adult human cardiomyocytes, are positioned in close proximity to the sarcoplasmic reticulum, an organelle involved in protein and lipid metabolism as well as Ca^2+^ transport [36,37,38]. Mitochondria also contribute to metabolic processes, such as β-oxidation, which generates acetyl-CoA from fatty acids, subsequently fueling the Krebs cycle. Through successive steps involving electron carriers, ATP synthase is ultimately activated. In addition to fatty acids, ATP production also relies on glucose, as well as ketone bodies, lactate, and amino acids—utilized to a lesser extent under resting conditions but increasingly under metabolic stress [39,40]. The ATP generated is essential for ion transport, calcium homeostasis, and actin–myosin interactions [41]. Cardiomyocytes respond to Ca^2+^ primarily via L-type calcium channels and ryanodine receptors. Here, subcellular localization is also critical. Invaginations of the sarcolemma form the T-tubule system, rich in ion channels, which lies adjacent to the sarcoplasmic reticulum. This arrangement facilitates excitation–contraction coupling, ultimately leading to contraction [42,43,44,45]. The sarcomere, the fundamental contractile unit of cardiomyocytes, comprises actin, myosin, tropomyosin, titin, and the troponin complex [46]. The contractile process is driven by conformational changes of these components [47]. Elevated intracellular Ca^2+^ levels activate the sarcoplasmic reticulum Ca^2+^ ATPase (SERCA), particularly the SERCA2a isoform in cardiomyocytes, which is regulated by phospholamban and sarcolipin. This ATP-dependent pump restores basal Ca^2+^ levels by translocating calcium back into the sarcoplasmic reticulum [48]. Maintaining ionic balance requires coordinated regulation of Ca^2+^ and Na^+^, mediated in part by the Na^+^/Ca^2+^ exchanger (NCX) and the Na^+^/K^+^ ATPase (NKA) [49].

#### 2.1.2. Disease-Related Alterations

Disease, much like development, induces reprogramming of cardiomyocyte metabolism [40]. Particular attention has been directed toward mitochondrial pathologies, including dysfunction of the electron transport chain and increased production of reactive oxygen species [50]. Cardiomyocyte loss may occur through processes collectively known as programmed cell death, such as apoptosis and autophagy. Certain stimuli, including ischemia, may trigger maladaptive programmed cell death, thereby contributing to disease pathogenesis [51].

Among conditions involving myocardial injury, myocardial infarction is of particular importance. The literature reports that a single infarct may result in the loss of 0.5 to 1 billion cardiomyocytes [52]. The dominant mechanism of cell damage during ischemia, similar to that observed with toxins, is oncosis, which is associated with cellular swelling. In addition to membrane defects, calcium overload is observed. The progression of these processes ultimately leads to remodeling and cardiac failure [53]. Structural alterations of cardiomyocytes are also evident in cardiomyopathies. In dilated cardiomyopathy, for instance, cardiomyocytes exhibit hypertrophy, calcium-handling abnormalities, fibrosis, and cell death, all contributing to the development of HF [54]. Truncating variants of the gene encoding the sarcomeric protein titin also play a crucial role in this context [55]. Hypertrophic cardiomyopathy is characterized by inefficient sarcomere function, excessive ATP consumption, and energetic stress. Patient myocardial tissue demonstrates mitochondrial oxidative abnormalities [56]. Similarly, disturbances of mitochondrial energy metabolism are observed in diabetic hearts, contributing to the pathogenesis of diabetic cardiomyopathy [57]. In arrhythmogenic right ventricular cardiomyopathy, genetic mutations affect intercellular junctions at the intercalated discs, leading to abnormal proteins. This cardiomyopathy is also associated with cardiomyocyte death, leading to fibrofatty replacement of myocardial tissue [58,59].

Cardiomyocyte abnormalities have additionally been reported in COVID-19 infection. A study by Siddiq et al. suggests that SARS-CoV-2–positive patients without prior cardiac disorders or comorbidities exhibit myocardial alterations, as indicated by elevated blood troponin levels. The same authors showed that viral infection of healthy cardiomyocytes, mediated by interleukins, results in cell enlargement and disorganization of contractile units [60]. Finally, HF is also characterized by cardiomyocyte loss. In this context, sarcolemmal restructuring has been reported [61]. The disease is associated with mitochondrial dysfunction, including substrate metabolism abnormalities, with metabolic differences influenced by coexisting obesity and type 2 diabetes [62]. Interestingly, Meddeb et al. described reduced calcium-induced myocyte tension and alterations in gene expression related to specific metabolic modifications in patients with HFpEF and severe obesity, with similar abnormalities also observed in HFrEF [63].

### 2.2. Direct Cell Transplantation

The concept of delivering cells to the heart with the expectation of improved function has been recognized for more than two decades [64]. Documented attempts include the use of stem cells directed toward differentiation into cardiac and vascular lineages. These processes are often described under the term remuscularization. An alternative mechanism is based on paracrine effects [65,66]. Researchers highlight MSCs, cardiac stem cells, and induced pluripotent stem cells (iPSCs) as strategies to substitute for myocardial loss and to promote reparative processes [67]. There are also reports on the application of embryonic stem cells (ESCs) in cardiac regeneration [68]. In the context of bone marrow–derived stem cells used in regenerative therapy studies, descriptions encompass not only MSCs but also, for example, hematopoietic stem cells (HSCs) [69]. Furthermore, investigations suggest that reprogramming fibroblasts into cardiomyocytes may represent another promising strategy [70]. Direct cell implantation constitutes one of the most dynamically developing domains within regenerative medicine. The literature emphasizes that the mode of delivery may represent a crucial determinant of therapeutic efficacy. Some authors argue that the outcome of therapy depends on this factor, thereby warranting careful consideration [70,71]. Approaches to cardiac cell delivery include intramuscular injection, intracoronary administration, endocardial injection, and intravenous infusion [72]. The latter is characterized as the simplest and least invasive method, whereas the former represents the most precise yet also the most invasive due to the necessity of thoracic access [68]. Such access is also required for the implantation of cardiac patches seeded with stem cells [73,74].

In this section, we discuss the groups of cells identified in the literature as candidates for regenerative cardiac therapies. In the concluding sections, we turn to their applications in preclinical studies and clinical outcomes.

#### 2.2.1. Skeletal Myoblasts

Historically, skeletal myoblasts are cited as the first cells employed for this indication. Skeletal myoblasts are classified as progenitor cells. Their therapeutic potential was based on the assumption that their contractile capacity would be preserved within the cardiac environment. An additional advantage was considered to be their adaptive capacity under ischemic conditions. Although the initial clinical studies suggested that these cells might represent a therapy capable of yielding significant improvements in cardiac function, subsequent investigations reported limited clinical efficacy. At present, research into their application has been discontinued due to adverse effects, most notably the induction of arrhythmias [75,76]. Moreover, it has been demonstrated that skeletal myoblasts are able to engraft within the myocardium; however, they lack the ability to express connexin proteins required for the formation of gap junctions, which are critical for appropriate electrical coupling. Consequently, their presence within the heart renders the tissue electrically unstable [77].

#### 2.2.2. Adult Stem Cells

The literature indicates that adult stem cells, such as bone marrow–derived cells, HSCs, and MSCs, demonstrate the capacity to differentiate into cardiomyocytes [70]. These cells are recognized for their abilities in self-renewal, tissue repair, and maintenance of structural integrity. Among them, MSCs are the most extensively studied, although significant data also exist for HSCs. They can be isolated from bone marrow, adipose tissue, and umbilical cord blood [78,79]. A principal advantage of this group of cells lies in their potential for autologous sourcing, thereby reducing the risk of immune rejection, as well as their relative availability. HSCs secrete a broad range of cytokines, growth factors, vascular endothelial growth factor (VEGF), and other molecules, supporting their role as promoters of cardiac tissue regeneration and angiogenesis [79]. However, their capacity to differentiate into cardiomyocytes is limited. As previously noted, their primary therapeutic effect is attributed to angiogenesis and the improvement of vascularization [80]. By contrast, MSCs attract considerable attention due to their strong immunomodulatory properties. They inhibit the proliferation of T lymphocytes, as well as B cells, natural killer (NK) cells, and dendritic cells. In addition, MSCs exhibit anti-inflammatory properties mediated through multiple mechanisms, such as downregulation of IL-1 expression and secretion of anti-inflammatory proteins [81]. They represent one of the most thoroughly investigated types of stem cells, with clinical studies consistently demonstrating their safety in therapeutic application [82]. Recent reports have also established the presence of cardiac progenitor cells (CPCs) within the adult heart. Under homeostatic conditions, they remain functionally inactive, yet in the setting of injury, resident CPCs can be activated. The literature describes their ability to differentiate into cardiomyocytes, vascular smooth muscle cells, and endothelial cells [83,84].

#### 2.2.3. Embryonic Stem Cells (ESCs)

ESCs are pluripotent cells derived from the human blastocyst. Their use, however, is associated with several obstacles, including ethical concerns, the risk of immune rejection, proarrhythmogenic effects, and tumorigenic potential [85]. Nonetheless, findings from studies employing these cells strongly indicate their regenerative capacity. Reported outcomes include improved cardiac function, enhanced angiogenesis, and reduced fibrosis, among other examples that underscore their therapeutic potential [68].

#### 2.2.4. Human Induced Pluripotent Stem Cells (hiPSCs)

hiPSCs represent a promising approach, as they are capable of adopting developmental pathways from all three germ layers. They are generated through the reprogramming of adult somatic cells using specific transcription factors. iPSCs offer the potential to give rise to ventricular, atrial, and nodal cardiomyocytes, as well as vascular smooth muscle cells and cardiac fibroblasts [71,86,87,88]. Their appeal lies in their relative ease of accessibility, favorable ethical profile, and the absence of immune rejection risk in the case of autologous transplantation [87]. However, an ongoing limitation concerns the immaturity of iPSC-derived cardiomyocytes, which may hinder their effective integration into defective myocardial tissue [89]. The study by Jebran et al. investigating allogeneic transplantation of engineered heart tissue containing iPSC-derived cardiomyocytes and stromal cells demonstrated favorable outcomes, including long-term retention for up to six months and an increase in the thickness of the target heart wall. These findings provided the foundational evidence supporting the approval of the first-in-human clinical trial [74].

### 2.3. Cellular Signaling in Regeneration

Cell-derived signals, known as the secretome, are biologically active compounds secreted by cells and utilized in intercellular communication [90,91]. These include, among others, growth factors, chemokines, cytokines, and exosomes [92]. These signals play a crucial role in cardiac muscle regeneration. Studies investigating the use of stem cells for repairing damaged myocardium have demonstrated that paracrine factors secreted by transplanted cells play a more significant role in regeneration than the differentiation of these cells into cardiomyocytes [10,93]. According to the paracrine hypothesis, stem cells secrete paracrine molecules that stimulate regenerative mechanisms such as cell proliferation and neovascularization [94,95]. The following sections will examine individual cell-derived signals and their impact on regeneration.

#### 2.3.1. Growth Factors

Growth factors secreted by CPCs are key cellular signals in regeneration due to their diverse actions [96]. One of these is VEGF. Hypoxia in the myocardium leads to increased VEGF expression [97]. The binding of VEGF to VEGFR (vascular endothelial growth factor receptor) on the surface of vascular endothelial cells activates VEGF/VEGFR signaling, which regulates angiogenesis in the damaged myocardium. VEGF/VEGFR signaling affects vascular permeability and stimulates endothelial cell proliferation and migration [97,98,99]. Other known growth factors include FGF (fibroblast growth factor) and HGF (hepatocyte growth factor). The FGF family comprises over 20 signaling proteins produced by various cell types, influencing multiple processes, including tissue regeneration [100]. Studies on the role of FGFs in cardiac repair have shown the effects of FGF1 and FGF2 on cardiomyocyte proliferation. The role of FGF9 in limiting cardiac fibrosis has also been demonstrated [101]. FGF, by binding to FGFR receptors, activates the FGF-FGFR signaling pathway, which regulates cardiomyocyte proliferation and exerts pro-angiogenic effects. For example, FGF1 activates the PI3K/AKT pathway, allowing cardiomyocytes to re-enter the cell cycle [102,103]. Studies on HGF have shown positive effects in repairing damaged myocardium due to its pleiotropic actions [104]. By binding to the tyrosine kinase c-Met, HGF stimulates cardiomyocyte proliferation and may also act pro-angiogenically by influencing VEGF expression [105,106].

#### 2.3.2. Cytokines

Paracrine-secreted cytokines play a key role in myocardial regeneration. Their roles have been demonstrated in angiogenesis, cardiomyocyte proliferation, and anti-apoptotic effects. These include, among others, TGF-β (Transforming Growth Factor-Beta) and interleukins IL-6 and IL-10. IL-10 has been shown to exert anti-inflammatory effects by reducing the secretion of pro-inflammatory cytokines [107]. Consequently, IL-10 can mitigate the harmful effects of pro-inflammatory cytokines in damaged myocardium. Another type of cytokine is chemokines, which control the migration of immune cells [108]. One of the most studied chemokines is CXCL12 (C-X-C motif chemokine 12). By binding to the CXCR4 receptor (chemokine receptor type 4), CXCL12 influences angiogenesis and promotes cardiomyocyte proliferation [109].

#### 2.3.3. Exosomes

MicroRNAs (miRNAs) are short non-coding RNA sequences that regulate regenerative processes in cardiomyocytes. Their length is about 22 nucleotides [110]. miRNAs have diverse effects through activation and inhibition of multiple pathways. For example, miRNAs involved in cardiomyocyte proliferation activate the YAP protein, which participates in transcription [111]. Well-characterized examples are miRNA-1, which regulates cardiomyocyte development, and miRNA-199-3p, which influences cell proliferation [112]. Another example is miRNA-21, which shows cardioprotective action by inhibiting apoptosis [113]. Exosomes have the potential to transform heart regeneration due to their low immunogenicity and ability to deliver multiple factors simultaneously. However, clinical application requires standardization of isolation methods and improvement of their specificity [114,115,116]. Table 1 presents examples of miRNAs, their origin, targets, and roles in cardiac muscle regeneration.

#### 2.3.4. Challenges in Cellular Signaling

Cell-derived signals form a network regulating key processes in regeneration, such as proliferation, angiogenesis, and apoptosis [122]. The use of cellular signals faces certain limitations. Many signals have a short half-life [123]. Cellular signals may also trigger undesirable effects, such as the chemokine CCL2. Studies suggest its positive effect on heart regeneration, but also its role in promoting atherosclerosis [124]. Despite promising research, current results remain insufficient. Further understanding of cellular signaling in cardiac muscle regeneration is necessary. Insights into these mechanisms open new therapeutic possibilities for treating heart diseases [125]. Developing cell-free therapies based on paracrine signaling will transform cardiac muscle regeneration by avoiding risks associated with cell-based treatments [67]. Table 2 summarizes cellular signal functions in cardiac muscle regeneration.

## 3. Research Findings

### 3.1. Experimental Studies Using Models and Animal Research

The most widely used animals for experimental models include mice and rats. The most common method for MI modeling in both rats and mice is surgical LAD ligation, which involves thoracotomy, exposure of the heart, and permanent or temporary ligation of the LAD artery. A reason that caused this method to become a current gold standard is that it produces infarcts that closely mimic human MI in terms of location, pathophysiology, and remodeling [128,129]. Another method used in animal models is chemical induction with agents such as isoprotenerol or doxorubicin being administered to induce diffuse injury of the myocardium. While it is easier to perform and is associated with higher survival rates, it less closely mimics the focal ischemia of human MI [130,131]. Another way is using a cold probe or electrocoagulation to create a localized infarct, though results may differ in tissue response compared to injury caused by ischemia [132]. Rodent MI models (mainly mice and rats) reliably reproduce key features of human MI, such as cardiomyocyte death, scar formation, and adverse remodeling, which is a value in terms of mechanistic studies and early-stage therapeutic screening [133]. They can recapitulate certain aspects of human autonomic regulation and heart rate variability post-MI [134]. Limitations include the fact that many regenerative therapies that show significant benefit in rodents fail to translate to humans, often due to differences in heart size, rate, immune response, and disease complexity. Rodent models typically lack the coronary atherosclerosis and plaque rupture seen in human MI, and the progression and kinetics of post-MI remodeling are much faster in rodents than in humans, therefore complicating interpretation of results [135,136].

Large animal models include primarily pigs, sheep, dogs, and non-human primates. Same as for rodents, the main method used is surgical coronary artery ligation. While allowing precise control over infarct location and size, the invasiveness is associated with higher perioperative risk. Another approach consists of using percutaneous techniques such as balloon occlusion or embolization (using microcoils or thrombus injection). Such an approach is less invasive and more clinically relevant and allows the researchers to induce both permanent and transient occlusions to model reperfused and non-reperfused MI [137,138,139]. Large animals, especially pigs and sheep, have heart size, coronary anatomy, hemodynamics, and electrophysiology closely resembling humans. Their size allows for clinically relevant delivery methods (e.g., catheter-based interventions, imaging, device testing) and long-term follow-up, which are not possible in rodents [140]. In addition, large animal models can reveal complications not seen in small animals, for instance, arrhythmias or immune responses, which is vital as it comes to improving the evaluation of safety before moving on to human trials [137,141]. As in rodent models, there are some limitations to bear in mind. Purchase, housing, and care are estimated to be around 30 to 90 times more expensive than for rodents. There are differences in breed, age, comorbidities, as well as higher perioperative mortality rates, rapid weight gain, and the need for specialized surgical and imaging expertise. What is more, ethical and regulatory constraints should be considered. Stricter animal welfare regulations and public concern, especially for animals such as dogs, can restrict use and increase administrative burden. While more similar to humans, large animals still do not fully capture the complexity of human cardiac disease, which includes polypharmacy and comorbidities [140,142].

#### 3.1.1. Cell Therapy

A substantial body of experimental research demonstrates that bone marrow-derived stem cells can improve cardiac function, reduce infarct size, and promote tissue repair after MI in both small and large animal models. In swine MI models, transplantation of bone marrow MSCs (BM-MSCs) led to significant improvements in LVEF and cardiac function, as measured by MRI and PET-CT. These benefits were associated with enhanced glucose metabolism and upregulation of metabolic and signaling pathways in the myocardium [143,144]. In porcine models, intramyocardial injection of bone marrow mononuclear cells (BM-MNCs) improved LVEF and reduced scar collagen density, with the greatest benefit seen when cells were delivered directly to the myocardium [145]. The primary therapeutic effects are attributed to paracrine mechanisms—BMSCs secrete factors. Major secreted factors include VEGF, FGF2, IGF-1, HGF, SDF1, and TGF-beta. They are responsible for the promotion of angiogenesis, reduction in apoptosis, and modulating inflammation, rather than direct differentiation into cardiomyocytes [144,145]. BMSC transplantation increases microvascular density, reduces fibrosis, and improves wall thickness in infarcted myocardium in both rat and rabbit models. Extracellular vesicles from BMSCs also promote angiogenesis and preserve cardiac function in rodent MI models [146,147,148]. Additional studies include the use of MSCs. MSC therapy leads to significant improvements in LVEF and overall cardiac function in rodent and large animal models (e.g., swine, rats) [144,149]. Meta-analyses report LVEF increases of 7–10 percentage points compared to controls, with the most pronounced effects seen in the first 8 weeks post-treatment. MSCs reduce infarct size and adverse ventricular remodeling, with the greatest benefits observed when delivered via transendocardial or intramyocardial injection [150,151].

Preclinical studies show that CPC therapy leads to significant improvements in cardiac function, reduction in scar size, enhanced angiogenesis, and favorable remodeling in animal models of HF. CPC therapy consistently increases LVEF in both small and large animal models. Meta-analyses report LVEF improvements of 10–14% in mice and 6–8% in large animals compared to controls [152,153,154]. CPC-treated animals show reduced left ventricular end-systolic and end-diastolic volumes, smaller scar size, and more viable myocardium, with benefits persisting for up to one year post-transplantation [155]. CPCs and their extracellular vesicles promote angiogenesis, increase cardiomyocyte survival, and reduce apoptosis, contributing to improved tissue repair [156].

Experimental data from animal studies demonstrate that induced pluripotent stem cell-derived cardiomyocytes (iPSC-CMs) can engraft, survive, and improve cardiac function after myocardial injury, with evidence of both remuscularization and paracrine effects. In non-human primates, allogeneic iPSC-CM transplantation after myocardial infarction led to robust graft survival for at least 12 weeks, electrical coupling with host myocardium, and significant improvements in cardiac contractile function. However, transient ventricular arrhythmias were observed post-transplantation [157]. In rats and mice, transplantation of human or murine iPSC-CMs improved LVEF, reversed adverse ventricular remodeling, and reduced myocardial fibrosis. Grafted cells survived for weeks, formed mature cardiac structures, and contributed to contractile function [158,159,160]. Long-term studies in rhesus macaques showed autologous iPSC-CMs could engraft and mature for up to 12 months without immunosuppression or teratoma formation [161].

Survival and engraftment rates of transplanted cells in cardiac therapy animal models are highly variable. Human iPSC-derived cardiac microtissues (CMTs) transplanted into immunosuppressed pigs after myocardial infarction showed clear engraftment onto native myocardium at 4 weeks post-transplantation, with superior retention compared to single-cell grafts [162]. In a mouse model using optimized iPSC-derived cardiomyocytes, on day 20, iPSC-cardiomyocytes demonstrated high engraftment and proliferation in infarcted mouse hearts, with engrafted cells surviving and maturing for at least 3–6 months [159]. In a rat model, co-transplanting micro-vessels with iPSC-cardiomyocytes led to a sixfold increase in cell survival. Approximately 80% of cells survived at 1 week and 60% at 4 weeks, with improved vascularization and graft maturation [163]. Another rodent model showed that engineered heart muscle grafts preserved up to 25% of transplanted cells at 12 weeks, with stable engraftment and no significant cell loss between weeks 2 and 12 [164]. A swine model using large cardiac muscle patches from human iPSC had an engraftment rate of 0.9% ± 1.8% at 4 weeks post-transplantation [165]. In the case of a study on rhesus macaques, already mentioned above, autologous iPSC-cardiomyocytes survived and matured for 6–12 months without immunosuppression, while allogeneic cells were rejected within 8 weeks. All in all, engraftment and survival rates in animal models of cardiac therapy range from 10 to 80% at early time points, with long-term survival possible using engineered tissues, immunosuppression, and autologous cells [161].

While offering the benefits of cellular therapy, preclinical studies also managed to point out a range of adverse effects. One of the most commonly reported is arrhythmia. Ventricular and atrial arrhythmias have been observed, particularly with skeletal myoblast and pluripotent stem cell-derived cardiomyocyte transplantation, due to poor electrical integration with host myocardium. In some studies, arrhythmia risk was not increased with MSC therapy, but this may depend on cell type and delivery. Immune rejection and inflammation can occur, especially with allogeneic or xenogeneic cells. Production of donor-specific antibodies has been reported in 19–34% of recipients of allogeneic cell therapies, though the clinical impact remains unclear. Local and systemic immune reactions, including fever and mild inflammatory responses, are common but usually transient [166,167]. Thromboembolic complications (e.g., myocardial or cerebral infarction, pulmonary embolism) have been reported after systemic or cardiac administration, though these are considered rare. As far as tumor formation is concerned, as of today, it remains a theoretical risk, especially with pluripotent stem cells, though it is rarely observed in preclinical cardiac studies. Other adverse events include infection, hemodynamic instability, and, rarely, death, but these are not consistently linked to cell therapy [73].

#### 3.1.2. Cell-Derived Signals

Robust preclinical evidence demonstrates that the stem cell secretome—comprising soluble factors and extracellular vesicles—can promote cardiac repair and regeneration after myocardial injury. Sustained delivery of human cardiac stem cells in rats after myocardial infarction preserved LVEF, reduced scar tissue and fibrosis, decreased cardiomyocyte hypertrophy, and increased vascular density. These effects were achieved via subcutaneous implantation of devices releasing the secretome, with evidence of secretome components reaching the heart and mediating repair [168]. Secretome from human amniotic fluid-derived stem cells (hAFS) improved the survival of cardiomyocytes under stress, promoted angiogenesis, and triggered the proliferation of CPCs in vitro. In vivo, both conditioned medium and extracellular vesicles from hAFS reduced scarring, enhanced cardiac function, and supported angiogenesis in mice post-infarction [169,170]. Injectable hydrogels loaded with stem cell secretome (from human adipose-derived stem cells) increased capillary density, reduced scar area, and improved cardiac function in rat models, with no significant inflammatory response [171,172].

Another revolutionary tool is exosomes derived from stem cells. They consistently promote cardiac repair after myocardial infarction in animal models by improving heart function, reducing fibrosis, enhancing angiogenesis, and protecting cardiomyocytes from apoptosis. Inhaled or systemically delivered stem cell exosomes in mice and swine models of myocardial infarction led to significant improvements in LVEF, reduced infarct size, and thickened ventricular wall, with enhanced cardiomyocyte proliferation and reduced fibrotic tissue [173,174]. Exosomes from hypoxia-conditioned MSCs and other sources deliver microRNAs (e.g., miR-125b, miR-25-3p, miR-214-3p) that suppress pro-apoptotic genes, reduce cardiomyocyte death, and promote survival after ischemic injury [175,176]. Exosomes stimulate new blood vessel formation in the infarcted myocardium, often by upregulating proangiogenic factors (e.g., via HIF-1α, SDF1, miR-205), leading to increased capillary density and improved tissue perfusion [177,178]. Some exosome preparations promote anti-inflammatory (M2) macrophage polarization, further supporting tissue repair and limiting adverse remodeling [179]. While exosome and secretome therapies show promise for cardiac repair, several key limitations have been identified in preclinical studies, affecting their translation to clinical use. One problem is the heterogeneity of the source and cargo. The content and therapeutic efficacy of exosomes/secretome depend on the donor cell type, cell state, and culture conditions, leading to batch variability and inconsistent results [96,180]. Furthermore, there is a lack of standardized isolation and purification. No consensus exists on optimal methods for exosome/secretome isolation, purification, and quality control, complicating reproducibility and scalability [15,181]. Last but not least, the mechanisms of cargo packaging and the biological activity of many exosomal components remain poorly understood [182]. Another problem is delivery and retention issues. Exosomes and secretome factors have a short half-life in vivo, often requiring repeated or high-dose administration for sustained effects. What is more, systemic delivery leads to off-target distribution (e.g., lungs, spleen), with low retention in the injured myocardium, reducing therapeutic efficacy [183,184]. The last important issue is dosing, safety, and efficacy uncertainties. The best dose, timing, and delivery route for maximal benefit and minimal risk are not yet established, and while generally less immunogenic than cells, exosomes, and secretome may still cause unintended effects or immune responses, especially with repeated dosing [15,96,183].

### 3.2. Clinical Evidence in Humans

Clinical guidelines regarding the efficacy of therapeutic methods are predominantly developed based on randomized clinical trials. The results obtained are subjected to rigorous analysis in order to quantify the average therapeutic effect within appropriately selected patient populations [185]. Consequently, such studies play a crucial role in evaluating both the safety and feasibility of therapeutic techniques, including those in cardiology. An analysis by Wulfse et al. revealed that only one-third of all registered clinical trials in the field of regenerative cardiology report clinically significant outcomes. Another limiting factor is that the majority of these trials remain incomplete. Furthermore, the authors emphasize substantial heterogeneity in study designs, which significantly complicates reliable comparisons of different interventional approaches and consequently delays the translation of these therapies into clinical practice [186]. Key challenges facing clinical trials include insufficient funding, the selection of appropriate endpoints, and the choice of suitable statistical methodologies, all of which are critical for the accurate analysis and interpretation of trial results [185].

#### 3.2.1. Cell Therapy

Cell-based therapies, including those utilizing stem cells, represent one of the most promising directions in regenerative medicine. These approaches offer significant potential for the repair and modification of disease-affected cells [187]. The CONCERT-HF study was among the first multicenter, double-blind, randomized clinical trials evaluating therapies involving MSCs and CPCs, both individually and in combination. The results demonstrated that transendocardial injection of autologous MSCs and CPCs, in any configuration, was safe. The use of CPCs was associated with an 80% reduction in the incidence of major adverse cardiac events (MACE) due to HF compared with the placebo group. Similar outcomes were observed in the cohort receiving combined MSC and CPC therapy. Furthermore, MSCs administered alone or in combination with CPCs led to improved scores on the Minnesota Living with Heart Failure Questionnaire (MLHFQ). In summary, combined therapy was associated with the most favorable clinical outcomes. Nevertheless, the authors emphasized persistent gaps in knowledge regarding the mechanisms underlying the therapeutic effects of these cells [188]. A Danish multicenter, double-blind, randomized clinical trial evaluated allogeneic adipose tissue–derived mesenchymal stromal cells (ASC) as an adjunct therapy in patients with chronic HF with reduced ejection fraction. Clinical improvement and enhanced quality of life were observed in the ASC group, though no statistically significant differences were noted between groups. Adverse events occurred but did not differ significantly between groups over a three-year follow-up. Overall, the study did not demonstrate that intracardiac ASC injections translate into improved cardiac function or myocardial structure, but it confirmed the safety of the approach [189]. The prospective five-year observational MYSTAR study analyzed outcomes following transendocardial administration of autologous bone marrow mononuclear cells (BM-MNCs), followed by intravenous coronary injections. This study demonstrated that the procedure was safe and led to improved cardiac function, particularly of the right ventricle, in patients with reduced ejection fraction post-myocardial infarction [190]. The ATHENA Trials, another double-blind study, aimed to evaluate the safety, feasibility, and efficacy of autologous adipose-derived regenerative cells (ADRCs) in patients with chronic ischemic cardiomyopathy. Unfortunately, the study was prematurely terminated due to major adverse cardiac events unrelated to ADRC therapy. However, partial assessments indicated a favorable change in VO_2_ max in the ADRC group, although no differences were observed in left ventricular function between groups. Improvements were noted in NYHA class, CCS class, and MLHFQ scores, which significantly favored the ADRC cohort. The authors highlighted the need for further research due to limitations, primarily the small sample size [191]. The TAC-HFT randomized trial also evaluated the safety and efficacy of MSCs and BM-MNCs in treating chronic ischemic cardiomyopathy in patients with ejection fractions below 50%. Over 12 months, the MSC group exhibited significant improvements in MLHFQ scores and the six-minute walk test distance. Infarct size relative to left ventricular mass decreased substantially in the MSC group, whereas changes in the BM-MNC and placebo groups were minimal. Regional myocardial function at the injection site improved most in the MSC group. No significant changes were observed in left ventricular volume or ejection fraction. The incidence of serious adverse events was lower in the experimental groups compared to controls. The authors concluded that these therapeutic approaches are safe but acknowledged limitations, such as small participant numbers, and recommended larger-scale studies [192]. Finally, the HEAL-CHF study, a single-center dose-escalation trial, enrolled patients with advanced HF. The therapy under investigation involved epicardial injection of human iPSC-CMs. The primary endpoint was to establish the safety of cell transplantation regarding persistent ventricular arrhythmias and tumor formation, while efficacy relative to a control group was a secondary endpoint. Although the study has been completed, results are not yet available [193].

#### 3.2.2. Cell-Derived Signals

The literature reports that it is possible to recapitulate the effects of stem cells through their secretome. One example is the FOCUS-CCTRN clinical study, which aimed to identify variable factors in MSCs among treated patients who demonstrated improvement in cardiac function. One such variable was the difference in MSC secretome levels [194]. Unfortunately, compared to cell-based therapies, this field still has a relatively limited body of research in humans. Currently, our knowledge relies solely on a single published study in this area, which has thus far reported its findings. Researchers from the SECRET-HF study described the first case of a patient presenting with severe drug-refractory left ventricular dysfunction due to non-ischemic dilated cardiomyopathy, classified as NYHA III, with a LVEF of 25%, who received an intravenous injection of extracellular vesicle–enriched secretome derived from cardiovascular progenitor cells. Six months after therapy, clinical improvement was observed, resulting in a reduction to NYHA class II. Additionally, echocardiographic parameters improved, no alloimmunization against the therapeutic product was detected, and the dose of administered diuretics was reduced. To date, this remains the only case reported from this study, which included 12 participants [195].

## 4. Discussion

Cardiac regenerative therapy bases its primary mechanism of action on both cells and paracrine signals derived from cells [196]. Both approaches represent the future of clinical practice, offering promising solutions for patients; however, each presents certain challenges and obstacles that must be considered when evaluating a given method [70].

Cell therapy is characterized by its comprehensive potential to efficiently repair damaged tissue or replace dead cells. Through transplantation, it provides rapid rescue for the failing heart, for instance, following a myocardial infarction [197,198]. The newly introduced cellular network additionally demonstrates continuous paracrine signaling, which further enhances therapeutic efficacy, ensuring a more effective and long-lasting regeneration of the myocardium [65,85]. Consequently, the transplanted tissue actively initiates neovascularization processes, which are responsible for proper angiogenesis of the damaged heart, a key factor for its complete reconstruction [199]. An additional advantage of this method is the ease of obtaining cells for therapy due to the broad diversity of potential sources. These range from autologous bone marrow-derived cells and adipose tissue-derived MSCs, to ESCs, cardiac stem cells (CSCs), and even hiPSCs, which can be derived from mature organisms and have the ability to differentiate into nearly all cell types of the adult body [11]. This diversity provides significant potential and flexibility, greatly increasing the chances of survival for cardiology patients. On the other hand, this therapeutic pathway also presents certain challenges, such as the high cost of treatment and the long, highly specialized process required to prepare cells for transplantation [200,201]. A major issue is the risk of transplanted tissue rejection or limited survival of cells in the hostile host environment [202,203]. Although new cells often exert anti-apoptotic and anti-inflammatory effects, which are highly beneficial for the diseased heart, these same properties may also contribute to the development of neoplastic diseases such as teratomas. The likelihood of this occurring depends on both the number and type of transplanted cells. Scientific reviews identify pluripotent ESCs as the most teratogenic, with a critical threshold of transplanted cells exceeding 10^5^ in the heart [204]. Eliminating these cells from therapeutic tissues can significantly reduce the risk of tumor development, thereby offering a safer therapeutic approach [205]. Cell therapies are also associated with the risk of arrhythmias caused by the excessive activity of new cells and immune reactions due to the non-specific origin of the transplanted tissue [206]. These complications may result in severe patient outcomes, ultimately reducing the overall effectiveness of the treatment [39]. Another concern is the ethical dilemma surrounding eugenics in transplanted cells. This debate primarily centers on ESCs, which are obtained from surplus embryos remaining after in vitro fertilization procedures. Some consider their use a violation of the integrity and autonomy of a developing human being. Significant discrepancies exist in legal regulations on this issue between different countries. For instance, in the United Kingdom, the use of ESC lines is allowed strictly for research purposes, with therapeutic or reproductive applications strictly prohibited. Conversely, in countries such as the United States and Italy, both research and any interventions involving embryos for the purpose of obtaining ESCs are strictly banned [207]. Furthermore, these cells carry a higher risk of tumor development or immune responses due to their developmental potential. Therefore, future advancements in cardiac regenerative therapies should be based on more detailed molecular and genetic analyses of these cells, or alternatively, focus on other pluripotent cells that raise fewer concerns regarding their widespread use [208].

Cell-based therapies form the foundation of regenerative medicine in cardiology, but paracrine mechanisms are the primary drivers of their effectiveness [65,169]. A prime example of this is exosomes, which integrate various cardiac structures along a shared pathway toward regeneration [10,209]. Intercellular signaling pathways are particularly notable for their lack of risk of immune reactions, as they rely on cells already present in the heart, focusing on the interactions and processes occurring between them [210]. As a result, issues related to transplant rejection, cell sourcing, or ethical concerns are eliminated [94]. The potential for tumor development associated with therapy is also reduced [211]. This approach limits the process of fibrosis in the heart by preventing scar formation and postoperative adhesions. Additional support for this phenomenon is provided by factors such as IGF-1, which reduces fibrosis and promotes angiogenesis, and PDGF, which modulates fibrosis, participates in scar formation and tissue repair, and also exerts anti-apoptotic effects [99]. As a result, vascularization is enhanced, further improving the survival chances of the diseased heart. The development of new blood vessels and remodeling of pre-existing vascular networks are largely driven by paracrine intercellular stimuli that activate surrounding cells. Factors such as VEGF and FGF, which play crucial roles in this process, can be delivered to the heart using biomaterials [212]. The importance of paracrine mechanisms is further supported by the role of SDF-1, a stromal-derived factor responsible for mobilizing and directing stem cells to the infarct site, leading to neovascularization and cardiac remodeling while improving overall cardiac function. Its increased expression may be associated with MSCs; however, even without direct cell transplantation, high levels of SDF-1 can contribute to cardiac repair, particularly in patients with coexisting diabetes [213]. Nevertheless, cell signaling-based therapy also has certain drawbacks that raise concerns about its application and remain a primary subject of future research. The unknown composition of cellular proteins involved in intercellular interactions complicates the precise modulation of these pathways [214]. Particular attention is given to secretomes, cellular structures responsible for molecule transport within tissues. The inability to control signaling pathways in vivo results in a low therapeutic quality standard and poor prognostic outcomes, which directly impact the therapeutic profile [215]. A major challenge lies in controlling the ongoing effects of administered factors, as this can lead to adverse outcomes, such as undesired vessel growth in areas other than the target site when delivering VEGF. Effectively delivering these factors specifically to ischemic regions, such as damaged heart tissue, is also problematic [212]. Short half-lives and stability issues of some molecules further complicate the determination of an appropriate therapeutic window. Factor dosage is also critical, as illustrated by the anti-fibrotic protein Sfrp2. When present at appropriate levels, Sfrp2 protects the heart from fibrosis; however, its deficiency or decreased levels may paradoxically promote fibrotic processes [94].

Combined strategies represent the most promising pathway for the development of cardiac regenerative therapies, as they maximize the benefits of both methods while minimizing their respective drawbacks. Integrating cell-based therapies with emerging biomaterial engineering and precise modulation of paracrine signaling pathways ensures greater safety and therapeutic effectiveness [216]. Manipulating signaling pathways such as Hippo-YAP, Notch, and Nrg-ErbB allows for the regulation of cardiomyocyte cell cycles, promoting their reactivation and enabling more efficient proliferation of these cells [217].

Cardiac regenerative therapies, whether based on cells or intercellular signaling, present numerous challenges to modern science [198]. Despite promising results, neither effective validation nor a breakthrough enabling their widespread application has yet been achieved. It remains difficult to determine which cells are the most suitable for transplantation, considering patient safety, prognosis, technological conditions, and preparation costs. Additionally, uncertainties persist regarding cell survival time, engraftment rates, arrhythmic complications, and immunological interactions. The precise mechanisms of paracrine signaling also remain poorly understood. It is essential to identify the optimal molecular profile, dosage, timing, and modulation methods for these signaling molecules [218,219,220]. Key limitations include the low retention and survival rates of transplanted cells in the new environment, as well as their poor integration with host cells. Potential solutions to these obstacles include genetic and epigenetic modifications of cells prior to transplantation, preconditioning strategies such as hypoxia or cytokine treatment, and biomaterial engineering approaches using 3D scaffolds, hydrogels, or microcarriers [221,222,223]. These measures aim to improve the delivery and engraftment of cells within the damaged heart tissue [224,225,226].

Unfortunately, these therapies also generate considerable controversy, making them a contentious issue within society. Transparent and open communication between clinicians and patients undergoing cell-based therapies is crucial. All experimental and novel approaches must undergo rigorous evaluation to ensure patients can provide fully informed consent. Unapproved, costly therapies lacking clinical evidence compromise patient safety and undermine public trust in emerging cardiac regenerative medicine. Therefore, it is vital to establish regulatory standards, oversight mechanisms, and equitable access to ensure the broad acceptance and clinical utility of these treatments [227].

Technological barriers and insufficient funding from governing bodies further hinder progress [228]. A lack of understanding or interest from sponsors—often due to perceived low profitability or distant prospects—frequently leads to the abandonment of promising research projects. A notable example is the discontinuation of a CD34+ cell-based therapy for refractory angina pectoris [229]. The costs of cell acquisition and therapy often far exceed the modest clinical improvements achieved, typically reflected in only slight enhancements of clinical parameters. However, dismissing positive results as insignificant is unjustified, as there is still substantial potential for meaningful improvement in failing myocardium function. Many scientists and clinicians mistakenly interpret minimal post-myocardial infarction improvements as evidence of overall inefficacy, overlooking the demonstrated benefits of these therapies for chronically failing hearts. Further clinical trial phases are essential to validate these findings [230].

These therapies also present numerous opportunities. Novel approaches—such as muscle patches created from classical pluripotent stem cells placed on fibrin scaffolds, collagen plates supporting cardiomyocyte mitosis and proliferation, or spray patches composed of platelet-fibrin gel containing paracrine factors that aid myocardial repair— have demonstrated excellent regenerative outcomes, underscoring the vast potential for further advancements in this field [215].

Future research should aim to develop both therapeutic strategies concurrently, with a particular focus on innovative biomaterials and tissue carriers to enhance the specificity of cardiac regenerative medicine. Standardized protocols must be established for cell selection, dosage determination, and administration guidelines, including the number and sequence of treatment cycles. Additionally, better clinical trial design is necessary to validate these therapies and assess their real-world hospital applicability, taking into account patient-centered outcomes such as hospitalization rates, recovery time, and long-term survival. It is critical to conduct large-scale, rationally designed, randomized studies to overcome current barriers and advance the field effectively [231,232,233]. Longer-term follow-up is also essential to capture broader clinical implications of these therapies [234]. Current reports unfortunately fail to demonstrate the efficacy of existing cell-based therapies, showing only minor, clinically insignificant improvements in LVEF, a key measure of cardiac performance [3,235]. However, the leading direction for future development should focus on paracrine signaling-based approaches, without which cardiac regeneration would not be fully achievable. Ongoing research should therefore emphasize the exploration of exosome-based strategies, as well as genetic and chemical modifications of cells to enhance their signaling pathways [236]. Pharmacokinetic modeling should also be considered to improve therapeutic outcomes. Interdisciplinary collaboration is crucial—bringing together clinical researchers, laboratory scientists, bioengineers developing cell acquisition and production methods, the pharmaceutical industry, and regulatory agencies—to secure adequate resources and ensure the safety and practical utility of emerging cardiac regenerative therapies [1,229]. 

A summary of the advantages and limitations of the subject is presented in Table 3.

## 5. Conclusions

Cardiac regenerative therapy, based on cells and the signals exchanged between them, represents a promising lifeline for patients with damaged or failing hearts. Cell-based therapies provide direct repair of the injured area or post-infarction scar, while also stimulating neovascularization; however, they carry risks such as graft rejection, arrhythmias, immune reactions, and tumor formation. Paracrine mechanisms, on the other hand, support angiogenesis, limit fibrosis, and improve cardiac function with minimal immunological risk, though they require further exploration for clinical validation due to the insufficient understanding of paracrine molecules and the challenges in modulating their activity and effects. Combining both approaches, utilizing advanced biomaterials and precise modulation of signaling pathways, maximizes benefits while minimizing limitations, offering the potential for meaningful improvement in cardiac function in patients with chronic HF. The effective development of these therapies requires future clinical trials founded on multicenter interdisciplinary collaboration and strict regulatory oversight to ensure safety, greater efficacy, and broad accessibility of innovative cardiac regenerative methods.

## Figures and Tables

**Table 1:** Examples of miRNAs, Their Targets and Roles in Cardiac Muscle Regeneration.

Example of miRNA	Origin	Target	Role in Regeneration	Source
miRNA-21	CPC-derived exosomes	PDCD4	Inhibition of apoptosis	[113]
miRNA-132	MSC-derived exosomes	RasGAP-p120	Angiogenesis Inhibition of apoptosis	[117]
miRNA-125b	MSC-derived exosomes	p53, Bak-1	Inhibition of apoptosis	[118,119]
miRNA-146a	CDC exosomes	IRAK1, TRAF6	Anti-inflammatory effect	[120]
miRNA-210-3p	MSCs-EVs	EFNA3	Angiogenesis	[121]

**Table 2:** Examples of Cellular Signal Functions in Cardiac Muscle Regeneration.

Cell-Derived Signal	Example of Function	Source
VEGF	Angiogenesis	[97,98,99]
FGF	Angiogenesis; Cardiomyocyte proliferation (FGF1, FGF2); Limitations of cardiac fibrosis (FGF9)	[100,101,102,103]
HGF	Angiogenesis; cardiomyocyte proliferation	[104,105,106]
IL-10	Anti-inflammatory action	[126,127]
CXCL12	Angiogenesis, cardiomyocyte proliferation	[109]

**Table 3:** Advantages and limitations of cell therapy and cell-signaling therapy in the treatment of cardiovascular diseases.

Cell Therapy		Cell-Signal Therapy	
Advantages	Disadvantages	Advantages	Disadvantages
direct differentiation into cardiomyocytes and vascular cells [237]	low engraftment and retention rates (typically <5%) [238]	cell-free approach eliminates safety concerns [239]	limited ability to replace lost cardiomyocytes [240]
paracrine signaling effects between transplanted cells promoting tissue survival [65]	limited cell survival in a hostile ischemic environment [202]	better stability and storage properties [226]	shorter duration of action requiring repeated dosing [239]
endogenous stem cell recruitment and activation [241]	potential for arrhythmias and immune rejection [203]	reduced immunogenicity compared to cells [210]	potential for pathological effects with high doses [239,242]
anti-apoptotic and anti-inflammatory effects [3]	tumorigenicity risk (especially with ESCs) [204]	targeted delivery of specific factors (VEGF, IGF-1) [243]	difficulty in achieving optimal factor combinations [242]
neovascularization and angiogenesis promotion [199]	high manufacturing costs and complexity [201]	lower manufacturing complexity [244]	limited clinical data compared to cell therapy [117]
proven safety profile in clinical trials [196]	ethical concerns with ESCs [207]	potential for repeated dosing [239]	risk of inflammatory reactions and adverse effects [117,212]
multiple cell types available (ESCs, iPSCs, MSCs, CSCs) [11]	regulatory challenges and lengthy approval processes [201]	modulation of endogenous repair mechanisms [245]	challenges in targeted delivery to cardiac tissue [246]
established delivery methods (intramyocardial, intracoronary) [238]	limited scalability for widespread clinical use [220]	reduced regulatory barriers [94]	less comprehensive repair compared to cellular approaches [117,246]

## Data Availability

The original contributions presented in this study are included in the article. Further inquiries can be directed to the corresponding authors.

## Abbreviations

The following abbreviations are used in this manuscript:

PDCD4	Programmed cell death 4
RasGAP-p120	Ras GTPase-activating protein
p53	Tumor protein p53
Bak-1	Bcl-2 antagonist killer 1
IRAK1	Interleukin-1 receptor-associated kinase 1
TRAF6	TNF receptor-associated factor 6
EFNA3	EphrinA3
CPC	Cardiac progenitor cell
MSC	Mesenchymal stem cell
MSCs-EVs	Mesenchymal stem cell-derived extracellular vesicles
CDC	Cardiosphere-derived cells
